# Interferon-α Abrogates Tolerance Induction by Human Tolerogenic Dendritic Cells

**DOI:** 10.1371/journal.pone.0022763

**Published:** 2011-07-27

**Authors:** Nicole Bacher, Edith Graulich, Helmut Jonuleit, Stephan Grabbe, Kerstin Steinbrink

**Affiliations:** Department of Dermatology, University Medical Center Mainz, University of Mainz, Mainz, Germany; Centre de Recherche Public de la Santé (CRP-Santé), Luxembourg

## Abstract

**Background:**

Administration of interferon-α (IFN-α) represents an approved adjuvant therapy as reported for malignancies like melanoma and several viral infections. In malignant diseases, tolerance processes are critically involved in tumor progression. In this study, the effect of IFN-α on tolerance induction by human tolerogenic dendritic cells (DC) was analyzed. We focussed on tolerogenic IL-10-modulated DC (IL-10 DC) that are known to induce anergic regulatory T cells (iTregs).

**Methodology/Principal Findings:**

IFN-α promoted an enhanced maturation of IL-10 DC as demonstrated by upregulation of the differentiation marker CD83 as well as costimulatory molecules. IFN-α treatment resulted in an increased capacity of DC to stimulate T cell activation compared to control tolerogenic DC. We observed a strengthened T cell proliferation and increased IFN-γ production of CD4^+^ and CD8^+^ T cells stimulated by IFN-α-DC, demonstrating a restoration of the immunogenic capacity of tolerogenic DC in the presence of IFN-α. Notably, restimulation experiments revealed that IFN-α treatment of tolerogenic DC abolished the induction of T cell anergy and suppressor function of iTregs. In contrast, IFN-α neither affected the priming of iTregs nor converted iTregs into effector T cells.

**Conclusions/Significance:**

IFN-α inhibits the induction of T cell tolerance by reversing the tolerogenic function of human DC.

## Introduction

The type 1 interferon IFN-α is naturally produced in viral and non-viral infections. It displays well-known antitumor activity [1], but even more important, multiple immunoregulatory activities have been described. Immune regulation by IFN-α includes effects on proliferation, survival and differentiation of T and B lymphocytes and cytoxicity of natural killer cells [2]. In addition, IFN-α promotes maturation, functional activity and motility of dendritic cells (DC) [3], [4]. Hence, multiple protocols have been established to promote the differentiation of DC by IFN-α in combination with various stimuli such as proinflammatory cytokines or TLR ligands as LPS [5], [6].

The effects of type 1 interferons are employed in therapies of severe viral infections, multiple sclerosis, myelo- and lymphoproliferative diseases as well as solid tumors like malignant melanoma [7], [8]. Melanoma represents the most malignant skin cancer with rising incidence during the last decades in white populations worldwide [9]. The high risk of metastasis accounts for the need of adjuvant therapy in early tumor stages. To date, IFN-α therapy represents the only effective adjuvant therapeutic approach against malignant melanoma as demonstrated in several studies. Nevertheless, effects of IFN-α on tumor-associated tolerance are not understood [10].

In malignant diseases, induction of tolerance by tumor-derived factors is one critical mechanism involved in tumor progression. The production of the immunosuppressive cytokine IL-10 by tumor cells themselves or by tumor-infiltrating immune cells is well known for malignant melanoma and other tumor entities [11], [12], [13], [14]. IL-10 modulates the biologic function of antigen presenting cells (APC) and of T cells [15]. In previous studies, we and others demonstrated that IL-10 induces a tolerogenic phenotype of DC (IL-10 DC) with impaired T cell stimulatory properties [16], [17], [18]. Furthermore, IL-10 DC have been shown to induce anergic regulatory CD4^+^ and CD8^+^ T cells (iTregs), which inhibit activated cytotoxic and helper T cells, resulting in a failure to kill melanoma cells [17], [19].

Human DC comprise a heterogeneous cell population and their functional potential depends on their origin, the cytokine microenvironment and cell/cell interactions. They are central in inducing immunity and in mediating immune tolerance in their role as professional antigen-presenting cells. Tolerogenic DC control the immune homeostasis and prevent the development of autoimmune diseases but are also involved in tumor development and progression [20].

We hypothesized that IFN-α may interfere with tumor-associated tolerance mechanisms. In the present study, we demonstrate that IFN-α abrogates the tolerogenic function of human IL-10 DC and thereby prevents the induction of T cell anergy and, subsequently the differentiation into suppressive iTregs. The efficacy of IFN-α treatment in cancer and infectious diseases may therefore be related to its capacity to break tumor-associated tolerance induction.

## Results

### IFN-α enhanced the maturation of tolerogenic IL-10DC

IFN-α was shown to promote maturation of DC in various settings [3], [4], [5], [6]. Here, the effect of IFN-α on the function of human tolerogenic DC was analyzed. We focussed on monocyte-derived tolerogenic IL-10 DC as inducers of anergic iTregs [16], [17].

**Figure 1:**
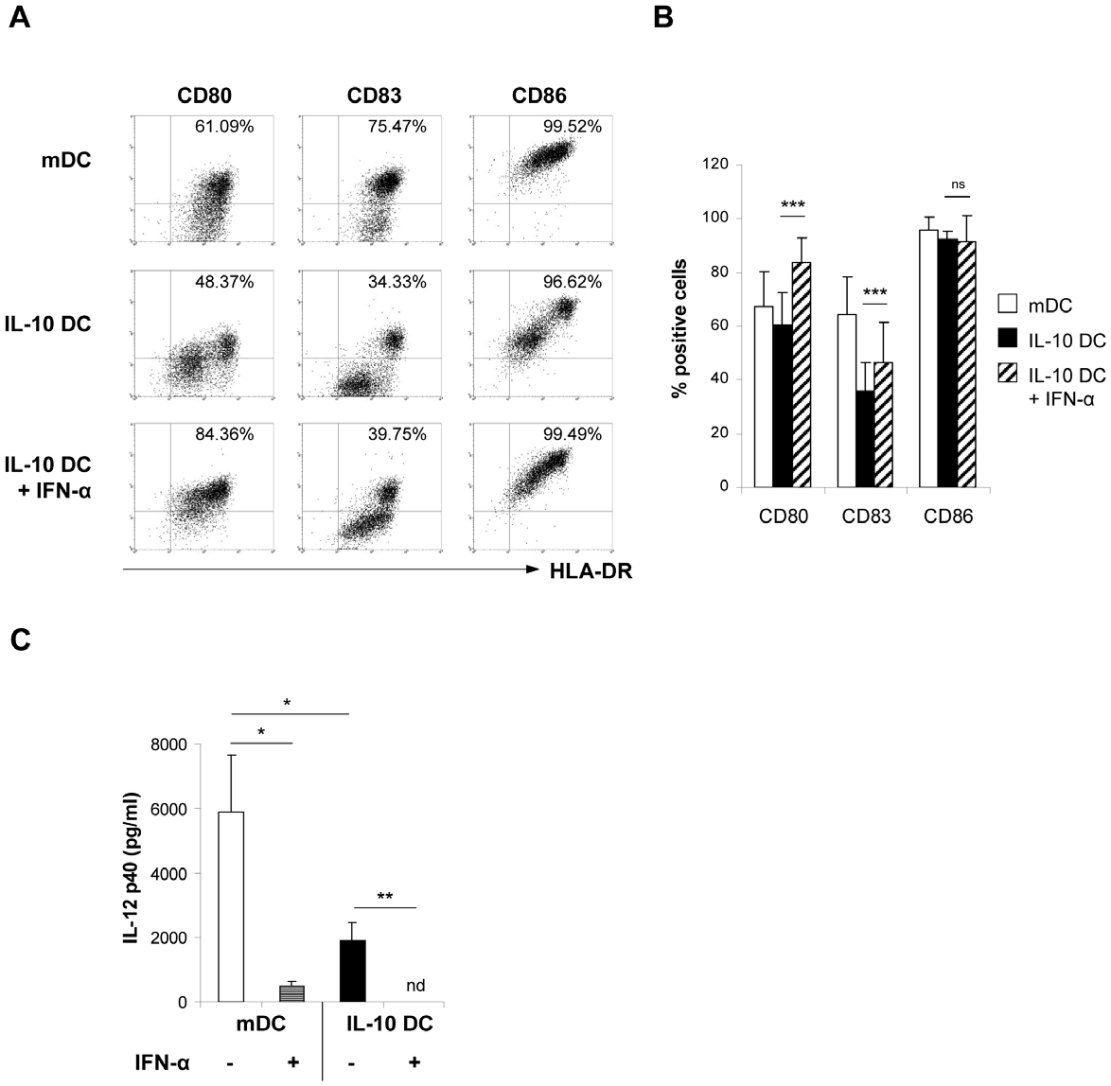
Immunophenotype of IL-10 DC after IFN-α treatment. (A, B) Immature DC were stimulated at day 5 of culture with a maturation cocktail (mDC) or a maturation cocktail supplemented with IL-10 either with or without 10^4^ U/mL IFN-α. (A) Dot plots of one representative experiment and (B) expression of surface molecules of DC of 6 independent experiments are depicted. (C) mDC and IL-10 DC and IL-10 DC +/−10^4^ U/mL IFN-α were generated as described in Materials and Methods. At day 7, supernatants were collected and production of IL-12 p40 was assessed by ELISA. Mean values +/− standard deviation (SD) of 4 independent experiments are shown; nd: not detected. * p<0.05; ** p<0.01; *** p<0.001, n.s. not significant.

First, in dose-dependent experiments concentrations of 10^2^–10^4^ U/ml IFN-α were identified to be effective in immune modulation without toxic effects (data not shown). To assess the effects of IFN-α on tolerogenic IL-10-modulated DC, IL-10 DC were incubated with IFN-α for two days during final maturation. We did not find an altered expression of MHC class II (HLA-DR) and CD86 molecules after IFN-α treatment (data not shown, Figure 1A, B). In contrast, expression of CD80 and CD83 was significantly augmented on IFN-α-treated IL-10 DC (Fig. 1A, B), demonstrating that IFN-α partially overcomes the tolerogenic properties of IL-10 DC during DC differentiation. No alteration of the phenotype of mDC was observed after IFN-α treatment during terminal maturation (data not shown).

Cytokine production of DC is critically involved in T cell priming and differentiation. Bioactive IL-12 represents an important cytokine by which DC stimulate outgrowth and activation of Th1/Tc1 cells, whereas the IL12p40 subunit also dimerizes with the p19 subunit to form IL-23 or mediates immunosuppressive functions [21], [22]. In our study, we found that IFN-α treatment of IL-10 DC as well as control mDC resulted in a significant reduction or complete inhibition of IL-12 p40 secretion, respectively (Fig. 1C). As previously demonstrated, IL-12p70 and IL-10 were not produced in significant amounts by mDC and IL-10 DC (data not shown) [16], [23]. Nor did IFN-α treatment induce secretion of IL-12p70 or IL-10 in both DC subsets (data not shown).

### IFN-α increased the stimulatory capacity of IL-10 DC

**Figure 2:**
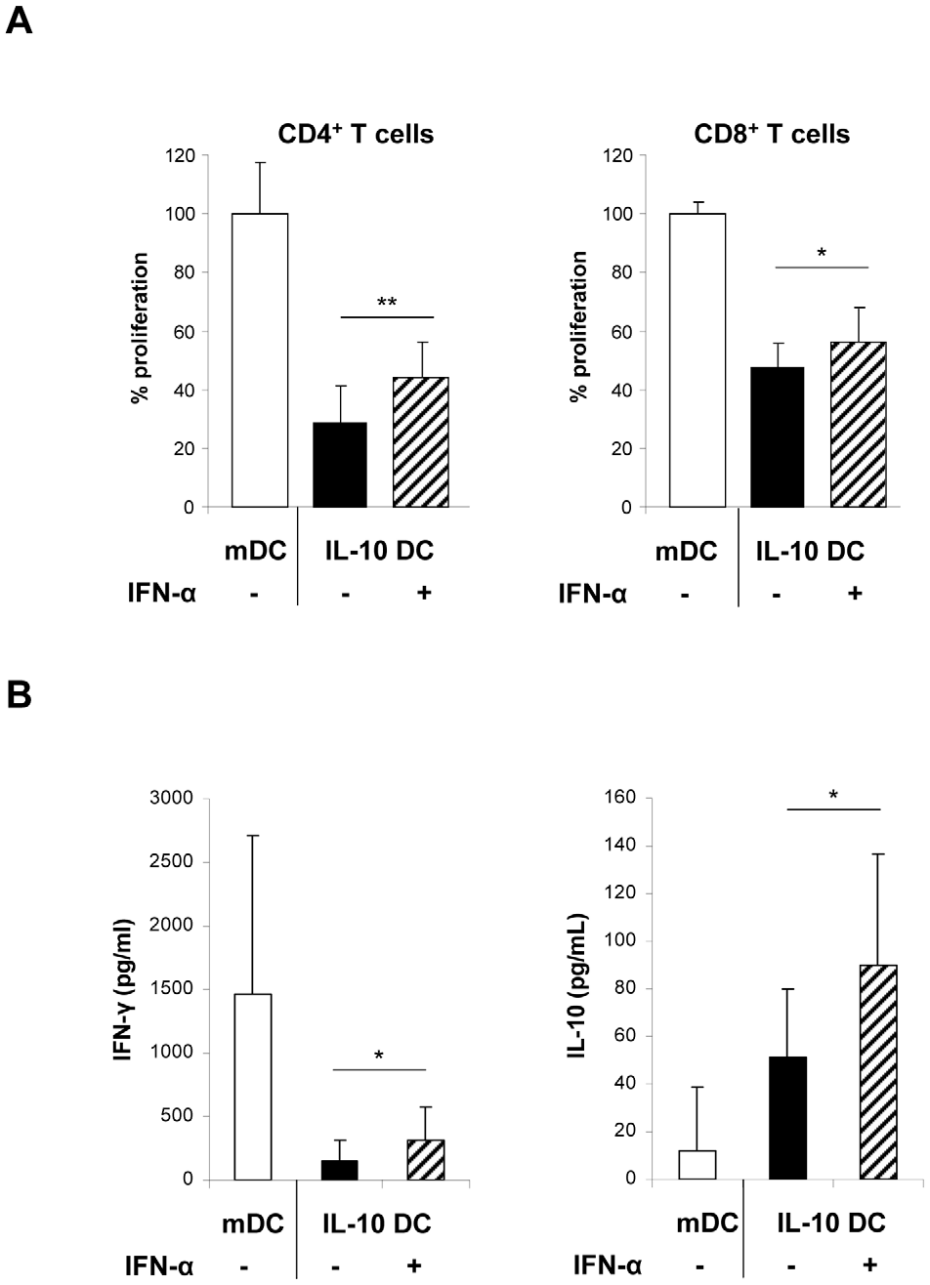
IL-10 DC generated in the presence of IFN-α induced an enhanced T cell activation. (A, B) CD4^+^ or CD8^+^ T cells were stimulated in primary culture with allogeneic mDC, IL-10 DC or IL-10 DC cultured with 10^4^ U/mL IFN-α. (A) T cell proliferation after restimulation was assessed as described and pooled data of 6 (CD4^+^ T cells) or 3 (CD8^+^ T cells) independent experiments are demonstrated. Proliferation is demonstrated in % +/−SD (normalized to 100% proliferation induced by control mDC). (B) IFN-γ and IL-10 production were detected in the supernatants of CD4^+^ T cells at day 5 of primary culture by ELISA. Mean values of cytokine levels in pg/ml +/− SD of 6 (IFN-γ) or 5 (IL-10) independent experiments are shown. * p<0.05; ** p<0.01; n.s. not significant.

IL-10 DC are weak stimulators of T cell proliferation compared to mDC [16], [24] (Fig. 2A). This phenomenon is essentially involved in differentiation of T cells into tolerogenic iTregs. In order to investigate the accessory function of IFN-α-treated DC, we performed a series of functional coculture experiments. Notably, IFN-α treatment significantly increased the T cell stimulatory properties of IL-10 DC, resulting in enhanced CD4^+^ and CD8^+^ T cell proliferation (Figure 2A).

Furthermore, IFN-α-DC provoked a significantly increased effector cytokine production of stimulated T cells. We observed enhanced levels of IFN-γ by co-cultured CD4^+^ T cells corresponding to the restored T cell proliferation (Fig. 2B). CD4^+^ T cells primed with IL-10 DC exhibited increased levels of the immunosuppressive cytokine IL-10 as compared to T cells incubated with mDC. A further increase in IL-10 levels was detected in supernatants of CD4^+^ T cells primed with IFN-α-treated IL-10 DC (Fig. 2B).

### IFN-α treatment of IL-10 DC prevented anergy induction in T cells

**Figure 3:**
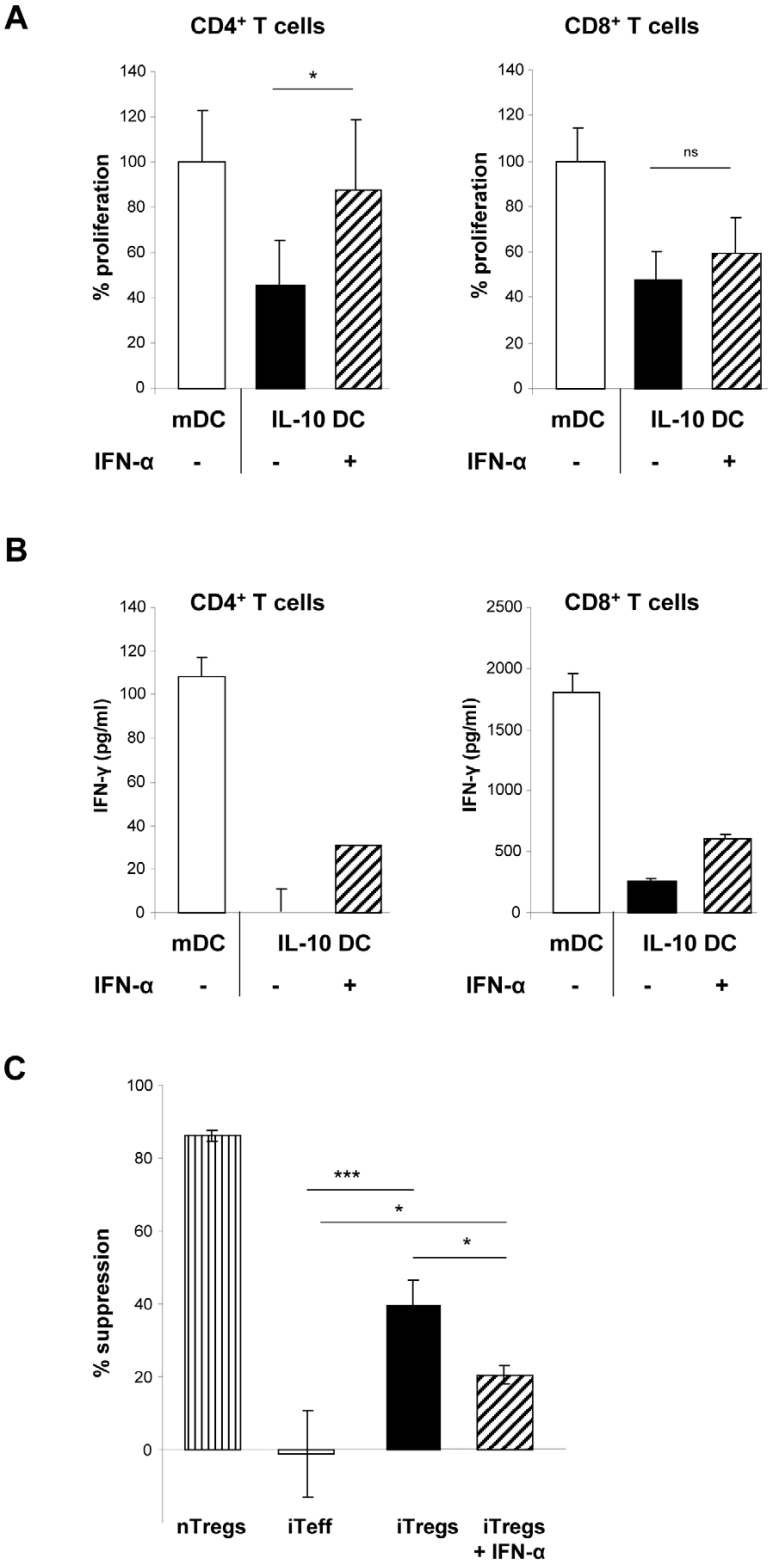
IFN-α treatment of IL-10 DC abolished suppressor function of iTregs. (A, B) CD4^+^ or CD8^+^ T cells were cultured in primary culture with allogeneic mDC, IL-10 DC or IL-10 DC treated with 10^4^ U/mL IFN-α, respectively. Restimulation experiments were performed with anti-CD3/anti-CD28. T cell proliferation is depicted in % (normalized to 100% proliferation induced by mDC). Mean values +/− SD of 4 (CD4^+^) or 3 (CD8^+^ T cells) independent experiments are shown. (B) Supernatants were obtained after 72 h and analyzed for IFN-γ production by ELISA. One representative experiment is demonstrated for CD4^+^ and CD8^+^ T cells, respectively. (C) Suppressor assay: CD4^+^ T cells primed with allogeneic mDC (iTeff), IL-10 DC (iTregs) or IL-10 DC treated with 10^4^ U/mL IFN-α (iTregs + IFN-α), respectively, or freshly isolated CD4^+^CD25^high^ nTregs were cocultured with CD4^+^CD25^low^ effector T cells and stimulated by 0.5 µg/mL anti-CD3 in the presence of irradiated PBMC. T cell proliferation was determined by [^3^H] thymidine incorporation. Suppressor activity is shown in proliferation of Teff + cocultured T cell population/poliferation of Teff ×100 in % +/− SD. One representative experiment of 3 is demonstrated. Results are shown in absolute cpm +/− SD for triplicates. * p<0.05; ** p<0.01; *** p<0.001, n.s. not significant.

To figure out whether IFN-α treatment of IL-10 DC modulates the capacity of tolerogenic DC to induce anergic T cells, we analyzed T cell proliferation in restimulation experiments. Proliferation of CD4^+^ and CD8^+^ T cells previously co-cultured with IL-10 DC was significantly inhibited, demonstrating the induction of an anergic T cell phenotype by IL-10 DC (Fig. 3A). However, IFN-α-treatment of IL-10 DC resulted in a significantly enhanced CD4^+^ T cell response, indicating abrogation of T cell anergy (Fig. 3A). Proliferation of CD8^+^ T cells primed with IFN-α-treated IL-10 DC was slightly but not significantly increased as compared to stimulation by control IL-10 DC (Fig. 3A).

Analyses of IFN-γ production after restimulation revealed that treatment of IL-10 DC with IFN-α resulted in increased IFN-γ production of CD4^+^ and CD8^+^ T cells, indicating a Th1/Tc1 skewing (Fig. 3B).

### Suppressor function of iTregs was abrogated after IFN-α treatment of tolerogenic IL-10 DC

Next, we assessed the effect of IFN-α on the suppressor function of anergic iTregs induced by IL-10 DC. Therefore, we analysed the suppressor capacity of iTregs on the proliferation of cocultured allogeneic CD4^+^CD25^low^ effector T cells (Teff). As shown in Fig. 3C, CD4^+^CD25^high^ naturally occurring regulatory T cells (nTregs) served as controls with high suppressor activity. As reported previously, iTregs induced by IL-10 DC, significantly suppressed the proliferation of cocultured Teff [17]. Importantly, IFN-α treatment during the generation of tolerogenic IL-10 DC significantly inhibited the suppressor function of iTregs primed by these DC, indicating that IFN-α annihilates the tolerogenic function of IL-10 DC and, therefore, the induction of anergic iTregs (Fig. 3C).

### IFN-α did not interfere directly with the induction or maintenance of T cell anergy

**Figure 4:**
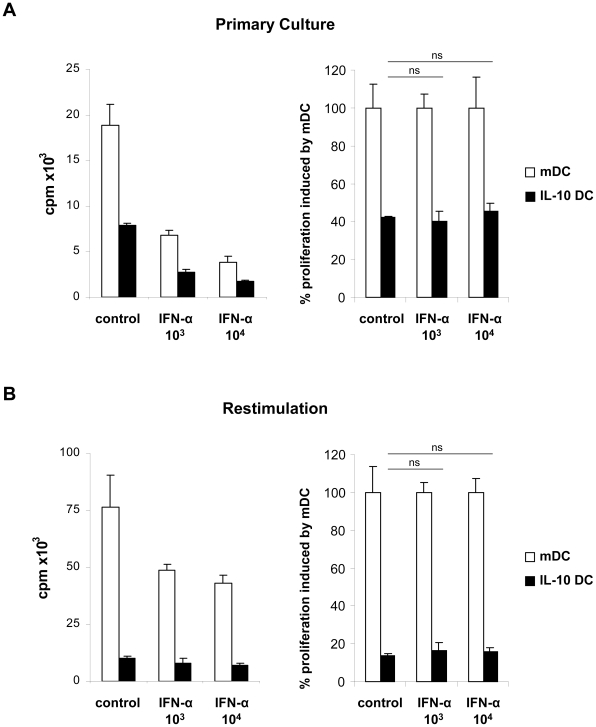
IFN-α did not interfere with priming or perpetuation of T cell anergy. (A) CD4^+^ T cells were cocultured with allogeneic mDC or IL-10 DC and incubated with concentrations of IFN-α as indicated. After 5 days, T cell proliferation was assessed by [^3^H] thymidine incorporation, results are depicted in absolute cpm +/− SD (left panel) and in percentages +/− SD (100% = T cell proliferation induced by mDC) (right panel). One representative experiment of 3 is shown. (B) CD4^+^ T cells primed with allogeneic mDC or IL-10 DC were restimulated with anti-CD3/anti-CD28 in the presence of increasing concentrations of IFN-α as indicated. T cell proliferation was detected after 72 hours of restimulation as described. Results are shown in absolute cpm +/− SD (left panel) and in percentages +/− SD (normalized to proliferation induced by mDC = 100%) (right panel). One representative experiment of 2 is demonstrated. n.s. not significant.

As shown previously, IFN-α exhibits inhibitory effects on T cell proliferation independent of the stimulating APC population or the activating agent [25], [26]. We addressed the question whether IFN-α is also capable to inhibit DC-T cell interactions required for induction of T cell anergy. In addition, we analyzed whether IFN-α abolishes the maintenance of the anergic T cell phenotype. For this purpose, T cells were incubated with IFN-α during primary cultures with DC or during restimulation experiments, respectively. As shown in Fig. 4A (left panel), IFN-α inhibited the T cell proliferation in primary cultures induced by both, mDC and IL-10 DC in a dose-dependent manner. Normalization with regard to the mDC-induced T cell proliferation ( = 100%) was calculated for each IFN-α concentration and medium control. The results revealed that the presence of IFN-α during DC-T cell interaction did not affect the induction of T cell anergy (Fig. 4A, right panel).

Similar results were obtained when the influence of IFN-α on the maintenance of the anergic T cell phenotype induced by tolerogenic DC was analysed. As expected, restimulation in the presence of IFN-α promoted a significant and dose-dependent reduction of effector T cell proliferation (induced by mDC). However, IFN-α did not affect the anergic state of CD4^+^ T cells (primed with IL-10 DC) as demonstrated for absolute values (Fig. 4B, left panel) or percentages normalized to effector T cell proliferation ( = 100%) (Fig. 4B, right panel). These results indicate that IFN-α does neither inhibit the induction of T cell anergy nor does it directly modulate the properties of anergic T cells induced by IL-10 DC.

## Discussion

In our study, we demonstrate that IFN-α abolishes the tolerogenic phenotype of human IL-10 DC and, thereby, prevents the induction of T cell anergy and regulatory T cells.

Tolerogenic DC can be induced by tumor- or immune cell-derived factors like IL-10 and are critically involved in tumor progression [11]. Previously, we have shown that addition of IL-10 during the generation of human DC induced a tolerogenic phenotype of DC which provoked antigen-specific tolerance in CD4^+^ and CD8^+^ T cells [16], [19], [27], [28]. The induced anergic iTregs inhibited activated effector T cell responses in an antigen-specific fashion and induced a melanoma antigen-specific anergy in CD8^+^ cytotoxic T cells resulting in failure of tumor lysis. In the present study, we show that IFN-α promotes the maturation of tolerogenic IL-10 DC. However, addition of IFN-α to IL-10 DC did not induce full maturation as triggered by supplementation of a maturation cocktail. Similar observations have been made by Santini et al. [3], demonstrating that IFN-α-induced generation of DC resulted in an increased, but incomplete expression of the maturation parameter CD83.

IFN-α is known to enhance the expression of the IL-12 receptor β1 and β2 chains on human T cells enhancing Th1/Tc1 immune responses, but, more dominantly, it negatively regulates IL-12 p40 and p70 production by APC [29], [30], [31]. Our study confirmed these results by demonstrating that incubation of mDC as well as of IL-10 DC with IFN-α strongly inhibited IL12p40 secretion. As previously reported, human monocyte-derived DC generated by the protocol used in this study did not produce significant amounts of IL-12p70 [23]. Here, we demonstrated that IFN-α treatment did not increase levels of IL-12p70 secretion. However, we found that IFN-α stimulation of IL-10 DC was followed by an enhanced CD4^+^ and CD8^+^ T cell proliferation and increased IFN-γ levels, indicating an amplified Th1/Tc1 cell response. If this effect is partially due to loss of the immunosuppressive function of the IL-12 p40 subunit or of the IL-23 heterodimer needs further evaluation [21]. In accordance to our data, IFN-α-treated human DC have been shown to act as effective APC in driving the development of Th1/Tc1 immune responses in vitro an in vivo and, more recently, to expand both Th1 and Th17 populations [3], [4], [32], [33].

In T cells, contradictory effects of IFN-α on proliferation, function and cell death were observed [34], [35], [36], [37], [38]. It was predominantly reported that IFN-α reduced the proliferative response of CD4^+^ T cells. The cytokine inhibits T cell proliferation by targeting the MEK/ERK-pathway and by reducing susceptibility and production of IL-2 [25], [26]. In our hands, we confirmed these results observing a dose-dependent impaired T cell proliferation in the presence of IFN-α in primary cultures or restimulation experiments. However, IFN-α did not restore the anergic and regulatory phenotype of iTregs induced by tolerogenic IL-10 DC, excluding T cells as target cells of IFN-α in our model of tolerance. Intriguingly, IFN-α treatment abolished the induction and suppressor function of iTregs by of tolerogenic DC. In line with our results, in SLE patients, the activation and function of naturally occurring CD4^+^CD25^+^FOXP3^+^ Tregs were inhibited by IFN-α, resulting in loss of tolerance [39], [40]. Unlike these results, IFN-α enhanced IL-10-induced differentiation of human functional T regulatory type I (Tr1) cells [41].

IFN-α is the cytokine with the longest record of use in clinical oncology [7]. Clinical treatment of melanoma patients with high dose IFN-α is well established and direct antitumor effects as well as modulation of the immune system are supposed to contribute to the beneficial effect of the therapy [2], [10]. In melanoma patients, a striking correlation between the clinical response and the development of autoimmune reactions has been demonstrated. Reports of a prospective study of high dose IFN-α regime linked the appearance of clinical and laboratory evidence for autoimmunity with improved outcomes as demonstrated for relapse-free and overall survival, assuming that IFN-α inhibits tolerance mechanisms [42], [43]. However, the impact of IFN-α on tolerance mechanisms had not been addressed in detail. To date, the effects of IFN-α on human tolerogenic DC are unknown, but there is evidence that the ability of DC to attract Tregs, and thereby exert inhibitory function, is imprinted during maturation of DC and prevented by IFN-α [44]. Similar results were reported for naturally occurring Tregs in a mouse model. This study demonstrated that IFN-α inhibits the suppressor capacity of murine nTregs via activation of APC [45]. These data are in line with our observations that IFN-α treatment abolishes the tolerogenic phenotype of human DC resulting in an inhibited induction and function of anergic iTregs.

In our study, we demonstrated that addition of IFN-α during the generation of tolerogenic IL-10 DC abrogated the tolerogenic phenotype, enabled sufficient T cell responses with enhanced Th1/Tc1-skewing and prevented from induction of anergic iTregs. Tolerogenic DC in the tumor environment as well as in the entire immune system substantially contribute to cancer progression and metastasis. Our study indicates that one pivotal effect of IFN-α treatment in melanoma patients may be the reversal of tolerogenic DC into stimulatory APC to enhance cancer control mechanisms.

## Materials and Methods

### Ethics statement

Human T cells and dendritic cells used in this study were generated from buffy coats of healthy volunteers with approval of the local ethics committee of Rhineland-Palatinate. A written informed consent was obtained from all participants.

### Culture medium

Iscoves MDM (PAA Laboratories, Pasching, Austria) supplemented with 2.5% autologous plasma was used for generation of DC. T cells were cultured and stimulated in X-VIVO20 (Lonza, Verviers, Belgum) supplemented with 0.5% autologous plasma.

### Antibodies

For flow cytometry analysis, antibodies against CD2 (6F10.3), CD14 (RM052), CD19 (J4.119), CD80 (MAB104), CD83 (HB15A) (all from Beckmann Coulter, Krefeld, Germany), CD86, (BU63; Serotec, Raleigh, NC), HLA-DR (Serotec), and mouse (MOPC-31-C, MOPC-173, and 27–35) and rat (A95-1, R35-95) subclass–specific isotypes (BD Pharmingen, Heidelberg, Germany) were used. As conjugated secondary reagents, FITC-conjugated goat anti–rat IgG (Biolegend, San Diego, CA) and PE-conjugated donkey anti–mouse IgG (Jackson Immunoresearch, Bar Harbor, ME) were used. For staining of magnetic-activated cell-sorter (MACS)–sorted T cells, FITC-conjugated CD4 (13B8.2) or CD8 (B9.11) and FITC-conjugated mouse IgG (697.1Mc7) (all from Beckmann Coulter) were used.

### Generation of DC

Human DC were generated as described [24]. Briefly, PBMC were isolated from buffy coats and non-adherent cells were cultured in 3 mL Iscoves MDM supplemented with 400 U/mL granulocyte-macrophage colony-stimulating factor GM-CSF) (Leukine; Bayer, Seattle, WA), 150 U/mL IL-4 (Immunotools, Friesoythe, Germany) and 2.5% autologous plasma. At day 5, nonadherent cells were collected, resuspended in Iscoves MDM supplemented with 2.5% autologous plasma, 400 U/mL GM-CSF and 150 U/mL IL-4, and additionally stimulated with 2.5 ng/mL IL-1β, 2.5 ng/mL TNF-α (Miltenyi Biotec, Bergisch-Gladbach, Germany), 25 U/mL IL-6 (Strathmann), and 0.5 µg/mL PGE2 (Minprostin; Cayman, AnnArbor, MI) to induce terminal differentiation of mature DC (mDC). Tolerogenic IL-10 DC were generated by addition of 20 ng/mL IL-10 (DNAX, Palo Alto, CA USA) to the cytokine cocktail for the last two days of culture. For IFN-α treatment, IL-10 DC were incubated with 10^4^ U/mL IFN-alpha (Roche, Basel, Switzerland) simultaneously to IL-10 during the last two days of culture.

### Preparation of T cell populations

CD4^+^ or CD8^+^ T cells were prepared from buffy coats using CD4- or CD8-MACS beads (MACS systems; Miltenyi Biotec) according to standard protocols (purity>95%).

### Induction and restimulation of iTregs and activated CD4^+^ or CD8^+^ effector T cells

DC and T cells were prepared as described above, and 5×10^5^ mDC or IL-10 DC +/− IFN-α were cocultured with 5×10^6^ CD4^+^ T cells per well in 3 mL X-VIVO20 supplemented with 0.5% autologous plasma and 2 U/mL IL-2 (Proleukin, Chiron GmbH, Ratingen, Germany) in 6-well plates for 5 days during primary culture to induce effector T cells (Teffs, mDC) and iTregs (IL-10 DC). Proliferation assays were performed with 0.5×10^5^ T cells per well and different numbers of allogeneic DC in X-VIVO20 supplemented with 0.5% autologous plasma and 2 U/mL IL-2 in 96-well plates and proliferation was measured at day 5 by [^3^H]-thymidine incorporation. After 3–5 days, restimulation experiments were conducted with T cells (0.5×10^5^/well) stimulated by 1 µg/mL anti-CD28 mAb (BD Pharmingen) in 96-well plates coated with 0.5 µg/mL anti-CD3 mAb (OKT3) in X-VIVO20 supplemented with 0.5% autologous plasma. After 72 h, proliferation was detected by [^3^H]-thymidine incorporation.

### Suppressor Assays

CD4^+^ CD25^low^ effector T cells (Teff) and CD4^+^CD25^+^ nTregs were isolated from buffy coats of healthy volunteers as described before [46]. Briefly, CD25^+^ cells were separated using limited amounts of CD25-MACS-beads (Miltenyi Biotec) resulting in CD25^high^ cells. Afterward, negative depletion of CD8^+^, CD14^+^ and CD19^+^ cells was performed using CD8-, CD14- and CD19-Dynabeads (Invitrogen, Darmstadt, Germany) (purity>85%). For preparation of CD4^+^CD25^low^ effector T cells (Teff), PBMC were depleted of CD25^+^ cells using CD25-MACS-beads and LD-columns (Miltenyi Biotec) and subsequently, CD4^+^ cells were purified as described above. For suppressor assays, 1×10^5^ Teff and 1×10^5^ CD4^+^CD25^+^ nTregs or CD4^+^ iTregs were co-cultured in X-VIVO20 and stimulated with 0.5 µg/mL anti-CD3 mAb in the presence of irradiated allogeneic PBMC (9000 rad, 90 Gy). Single cultures of 1×10^5^ Teff, nTregs or iTregs served as controls. After 96 h proliferation was measured by [^3^H]-thymidine incorporation.

### Cytokine analysis

For assessment of cytokine production, supernatants were collected at day 8 of DC culture, 120 h after coculture of DC with T cells or 72 h after restimulation and stored at −70°C. Amounts of IFN-γ and IL-10 were assessed by ELISA using commercially available antibodies and standards according to the manufacturer's protocols (Immunotools). Levels of IL-12p40 were measured by ELISA using BD OptEIA Sets (Becton Dickinson).

### Statistical analyses

Statistical significances of differences between experimental groups were evaluated using the paired or unpaired Student's *t* test or the 1-way ANOVA and Tuckey's Multiple Comparison test and the GraphPadPrism 5 softwarepackage (Graphpad, La Jolla, California). *p* values of 0.05 or less were considered significant.
